# SARS-CoV-2 Infection among Pregnant and Postpartum Women, Kenya, 2020–2021

**DOI:** 10.3201/eid2709.210849

**Published:** 2021-09

**Authors:** Nancy A. Otieno, Eduardo Azziz-Baumgartner, Bryan O. Nyawanda, Eunice Oreri, Sascha Ellington, Clayton Onyango, Gideon O. Emukule

**Affiliations:** Kenya Medical Research Institute, Kisumu, Kenya (N.A. Otieno, B.O. Nyawanda);; Centers for Disease Control and Prevention, Atlanta, Georgia, USA (E. Azziz-Baumgartner, S. Ellington);; Ministry of Health, Siaya, Kenya (E. Oreri); Centers for Disease Control and Prevention, Kisumu, Kenya (C. Onyango);; Centers for Disease Control and Prevention, Nairobi, Kenya (G.O. Emukule)

**Keywords:** 2019 novel coronavirus disease, coronavirus disease, COVID-19, severe acute respiratory syndrome coronavirus 2, SARS-CoV-2, viruses, respiratory infections, zoonoses, COVID-like illness, influenza, pregnancy, postpartum, infants, Kenya

## Abstract

We determined incidence of severe acute respiratory syndrome coronavirus 2 and influenza virus infections among pregnant and postpartum women and their infants in Kenya during 2020–2021. Incidence of severe acute respiratory syndrome coronavirus 2 was highest among pregnant women, followed by postpartum women and infants. No influenza virus infections were identified.

Information about the incidence of severe acute respiratory syndrome coronavirus 2 (SARS-CoV-2) infection among hospitalized pregnant women is available (*1*), but information about incidence among pregnant women in the community is not. We therefore quantified the incidence of symptomatic laboratory-confirmed SARS-CoV-2 and influenza infections among pregnant and postpartum women and their infants in Kenya during 2020–2021. The study was reviewed and approved by the Kenya Medical Research Institute Scientific and Ethics Review Unit (KEMRI SSC. 2880) and the Centers for Disease Control and Prevention (CDC) Institutional Review Board (CDC protocol 6709; 45 C.F.R. part 46; 21 C.F.R. part 56). All participants provided written consent.

We adapted an ongoing prospective multiyear influenza mother/baby cohort to include SARS-CoV-2 testing (*2*). Pregnant women at <31 weeks of gestation who were seeking prenatal care in Siaya County, Kenya, were approached for enrollment. Those who provided informed consent completed a survey about their demographics and antenatal history and were tested for HIV infection. Women were then phoned or visited at home once weekly until delivery and through their postpartum period, together with their infants, for 6 months to identify coronavirus disease (COVID-19)–like illness (CLI) as defined by CDC (*3*). Those reporting CLI underwent nasopharyngeal and oropharyngeal swabbing at study clinics. Specimens were tested for SARS-CoV-2 and influenza viruses by real-time reverse transcription PCR at the KEMRI laboratory in Kisumu, Kenya. 

During May 2020–February 2021, KEMRI staff approached 1,056 pregnant or postpartum women and enrolled 1,023 (97%). Half of enrolled women had primary school education, and 40% ran small businesses. A total of 180 (18%) were HIV infected, of which 177 (98%) were receiving antiretroviral medication. A total of 116 (11%) were vaccinated against influenza. Each of 3 women had hypertension, diabetes mellitus, or tuberculosis. 

As of February 2021, staff had followed 886 pregnant women, who contributed 2,786 person-months, and 695 postpartum women and infants, who contributed 2,264 person-months (some women were represented in both groups). CLI developed in 274 (31%) pregnant women (348 episodes), 133 (19%) postpartum women (162 episodes), and 231 (33%) infants (277 episodes). Swab samples were collected within <10 days of illness from 58%; positive SARS-CoV-2 results were obtained for 12/200 (6%) pregnant women, 4/100 (4%) postpartum women, and 2/200 (1%) infants. None had positive influenza virus test results. The most common clinical manifestations of COVID-19 among pregnant and postpartum women were cough (16/16; 100%), runny nose (10/16; 63%) and headache (10/16; 63%). Cough was identified for each of the 2 SARS-CoV-2–infected infants (Table). The rate of SARS-CoV-2 infection rapidly increased during follow-up. In the population tested, the cumulative incidence of SARS-CoV-2 infection per 1,000 person-months was 4.3 (pregnant women), 1.8 (postpartum women), and 0.9 (infants) (Figure).

**Table:** Characteristics of pregnant and postpartum women and their infants with laboratory-confirmed severe acute respiratory syndrome coronavirus 2 infection, Kenya, May 2020–February 2021*

Characteristic	Values
Women, n = 16	
Days from onset to swabbing, mean (SD)	2.6 (1.9)
Care-seeking from onset, d	
<2	11 (68.8)
>2	5 (31.3)
Self-reported symptoms	
Fever in past 48 h	2 (12.5)
Measured fever >38.0°C	2 (12.5)
Cough	16 (100)
Shortness of breath	1 (6.3)
Runny nose	10 (62.5)
Headache	10 (62.5)
Muscle/ joint pain	2 (12.5)
Antimicrobial medication	16 (100)
Infants, n = 2	
Days from onset to swabbing, mean (SD)	2.5 (2.1)
Care-seeking from onset, d	
<2	1 (50.0)
>2	1 (50.0)
Clinical signs reported by mother	
Fever in previous 48 h	1 (50.0)
Measured fever >38.0°C	1 (50.0)
Cough	2 (100)
Runny nose	1 (50.0)
Diarrhea	1 (50.0)
Antimicrobial medication	2 (100)

*Values are no. (%) unless otherwise indicated.

**Figure:**
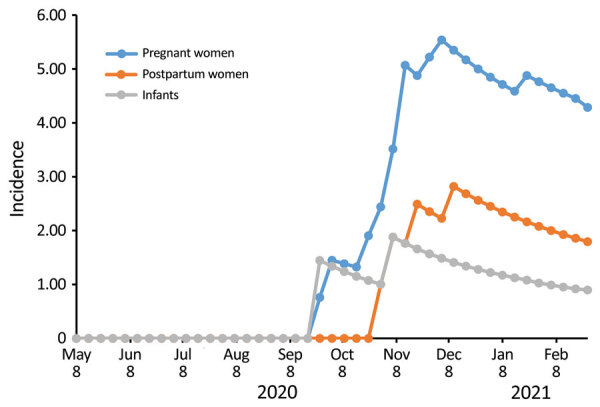
Incidence (cases/1,000 person-months) of severe acute respiratory syndrome coronavirus 2 infection among pregnant and postpartum women, Kenya, 2020–2021.

CLI occurred in 19%–33% of participants, of which a small percentage had laboratory-confirmed SARS-CoV-2 infection. The incidence of SARS-CoV-2 infection in this population, however, was rapidly rising during the study period. No influenza viruses were identifiable during the historic influenza epidemic period (*4*). SARS-CoV-2 rates seemed higher among pregnant women, then postpartum women, and lowest among infants. 

A study limitation is our inability to exhaustively assess symptoms of CLI among infants (e.g., headache, sore throat, loss of taste and smell) because we relied on the mothers’ reports. This limitation would potentially underestimate the burden of COVID-19 among infants. In addition, we did not quantify asymptomatic and mildly symptomatic infections that might have been missed. However, we plan to test acute-phase and convalescent-phase serum, cord blood, and placentas to identify asymptomatic infections and explore whether risk for SARS-CoV-2 infection truly differs. 

In summary, our findings suggest a higher burden of COVID-19 during pregnancy. These results highlight the potential benefit of prioritizing COVID-19 vaccination for pregnant women.
